# Hyaluronic Acid Hydrogel Containing Resveratrol-Loaded Chitosan Nanoparticles as an Adjuvant in Atopic Dermatitis Treatment

**DOI:** 10.3390/jfb14020082

**Published:** 2023-01-31

**Authors:** Raffaele Conte, Ilenia De Luca, Anna Valentino, Pierfrancesco Cerruti, Parisa Pedram, Gustavo Cabrera-Barjas, Arash Moeini, Anna Calarco

**Affiliations:** 1 AMES Group Polydiagnostic Center, Via Padre Carmine Fico, 24, 80013 Casalnuovo di Naples, Italy; 2 Research Institute on Terrestrial Ecosystems (IRET)—CNR, Via Pietro Castellino 111, 80131 Naples, Italy; 3 Institute of Polymers, Composites and Biomaterials (IPCB)—CNR, Via Campi Flegrei, 34, 80078 Pozzuoli, Italy; 4 Lehrstuhl für Brau- und Getränketechnologie, School of Life Sciences, Technische Universität München (TUM), 85354 Freising, Germany; 5 Unidad de Desarrollo Tecnológico (UDT), Universidad de Concepción, Avda. Cordillera No. 2634, Parque Industrial Coronel, Coronel 4191996, Chile

**Keywords:** hyaluronic acid, atopic dermatitis, resveratrol, chitosan, hydrogel, nanoparticles, antioxidant, anti-inflammatory

## Abstract

Atopic dermatitis (AD) is a common disease-causing skin inflammation, redness, and irritation, which can eventually result in infection that drastically impacts patient quality of life. Resveratrol (Res) is a natural phytochemical famed for its excellent anti-inflammatory and antioxidant activities. However, it is poorly bioavailable. Thus, a drug delivery system is needed to enhance in vivo bioactivity. Herein, we report the preparation of hyaluronic acid (HA) hydrogels containing resveratrol-loaded chitosan (CS) nanoparticles, their physicochemical analysis, and their potential therapeutic effects in the treatment of AD. Positively charged CS nanoparticles prepared by tripolyphosphate (TPP) gelation showed sizes ranging from 120 to around 500 nm and Res encapsulation efficiency as high as 80%. Embedding the nanoparticles in HA retarded their hydrolytic degradation and also slowed resveratrol release. Resveratrol released from nanoparticle-loaded hydrogel counteracted the oxidative damage induced by ROS generation in TNF-α/INF-γ-treated human keratinocytes (HaCaT) used as an AD in vitro model. Moreover, pre-treatment with Res@gel reduced secretion and gene expression of proinflammatory cytokines in HaCaT cells. The physicochemical analysis and in vitro assay confirmed that the formulated hydrogel could be considered an efficient and sustained resveratrol delivery vector in AD treatment.

## 1. Introduction

Atopic dermatitis (AD) is a well-known skin disorder with the main symptoms of pruritus, erythema, and skin lesions. Scratching due to AD makes the skin more susceptible to infections [1]. Steroidal drugs have been widely applied as antipruritic agents because of their anti-inflammatory properties. However, their severe side effects, such as petechiae, telangiectasia, muscle atrophy, and liver damage, limit their long-term application [2,3]. Therefore, the quest for a suitable antioxidant and anti-inflammatory agent to replace it with steroidal drugs made researchers shift their path to natural additives. The latter has been traditionally used mainly for wound healing [4,5,6,7]. However, for decades, scientists have generally invested in synthesis-based drugs in developing medical technologies. Thanks to the growing tendency toward bio-based and biocompatible materials, natural compounds have regained a significant contribution to medical and pharmaceutical science [5,6,8].

Resveratrol (Res), a natural polyphenol with antioxidant, anti-inflammatory, antiaging, cardioprotective, and neuroprotective properties, is one of the natural compounds beneficial for the treatment of AD [9,10]. Antipruritic characteristics of Res, which result from its capacity to neutralize reactive oxygen species (ROS) and attenuate interleukin expression, have been widely investigated [11]. However, Res is poorly water soluble and requires suitable formulations to increase its bioavailability. In the past decades, hydrogels have been widely used as dermal scaffolds and drug carriers due to their ability to mimic the extracellular matrix (ECM) of natural tissue [11,12,13]. Hydrogels present several properties, such as biocompatibility, porosity, mechanical strength, and high sensitivity to physiological environments with sufficient flexibility [14,15]. Due to its pharmacological properties, hyaluronic acid (HA) has been extensively used in pharmaceuticals and cosmetics since it is fundamental in many cellular and tissue functions [16]. Depending on its molecular weight and physical or chemical modifications, HA is available in various forms, including viscoelastic solutions, soft or stiff hydrogels, electrospun fibers, non-woven meshes, microporous and fibrillar sponges, flexible sheets, and nanoparticulate fluids [16]. Topical formulations based on drug-loaded nanoparticles in HA hydrogel have been reported as a valid approach for wound healing applications thanks to their improved effect on drug percutaneous transport across the stratum corneum barrier [17,18]. Dehkordi et al. developed a nanocrystalline (CNC)-reinforced HA-based composite containing chitosan nanoparticles loaded with growth factors as an effective wound dressing [19]. In vivo results demonstrated the ability of the synthesized composite to induce an almost full wound closure and total re-epithelialization with lower inflammatory reaction and improved formation of granulation tissue respect to the normal saline-treated wounds. In another study, Zhou and colleagues prepared a topical formulation for skin wound healing combining curcumin-loaded polycaprolactone/polyethylene glycol nanomicelles (PCEC/Cur) and HA (Cur/HA) [20]. Data demonstrated that after 14 days, Cur/HA leads to 96 ± 3% of wound healing in full-thickness skin damage in rats. Furthermore, in the Cur/HA group, immunohistochemical assays indicated a rapid re-epithelialization of hair follicles as well as improved angiogenesis and the healing of the wound surface.

Herein, Res-loaded chitosan nanoparticles (Res-NPs) were prepared and dispersed in hyaluronic-acid-based hydrogel (Res@gels) to obtain a topical formulation as an adjuvant in atopic dermatitis treatment. Chitosan (CS), a linear cationic polysaccharide of β-1,4-glucosamine and N-acetyl glucosamine, due to its biodegradability, biocompatibility, non-toxicity, and antimicrobial properties, represents a promising candidate as a drug carrier in biomedical and pharmaceutical fields [21,22,23,24,25]. Res-NPs were obtained by ionotropic gelation using sodium tripolyphosphate (TPP) as anionic crosslinking agents [26,27]. The Res-NPs were characterized regarding size, zeta potential, and ability to stabilize resveratrol. The most promising formulation was dispersed into HA gel, and rheological behavior and Res release were evaluated. Res released from Res@gels was able to protect TNF-α/INF-γ-induced human keratinocytes (HaCaT) from ROS damage and to reverse the secretion of various proinflammatory cytokines and chemokines. Hence, the formulated hydrogel is proposed as an efficient and sustained Res delivery system in inflammatory skin disease therapy such as atopic dermatitis.

## 2. Materials and Methods

### 2.1. Materials

Resveratrol (Res, >98% purity), medium-molecular-weight chitosan (CS, 50,000–190,000 Da, 75–85% deacetylated, viscosity <200 mPa·s, 1% in acetic acid), lactic acid (DL-Lactic acid, powder), sodium tripolyphosphate (TPP, technical grade), fluorescein isothiocyanate (FITC), 3,3′,5,5′-Tetramethylbenzidine (TMB), thiobarbituric acid (TBA), dexamethasone (#D4902, DEX), dichloro-dihydro-fluorescein diacetate (DCFH-DA), Cell Counting Kit-8 (CCK-8), and HA in the form of sodium hyaluronate from *Streptococcus zooepidemicus* were purchased from Sigma Aldrich (Milan, Italy) and used as received. When not specified, all other reagents used in the experiment were of analytical grade and purchased from Sigma Aldrich (Milan, Italy).

### 2.2. Preparation and Physicochemical Characterization of Res-Loading Nanoparticles (Res-NPs)

The Res-loaded nanoparticles (Res-NPs) were synthesized by modest modifications of the ionotropic gelation method using three different chitosan concentrations (0.1%, 0.5%, and 1% *w*/*w*) [28]. Valentino et al. [26] reported that chitosan was solubilized using 1% (*v*/*v*) lactic acid overnight at room temperature. Then, TPP (5 mg/mL) and Res (10 mg) were dissolved in ethanol and slowly added to CS solution under stirring (750 rpm) for 1 h to allow complete interaction and collected by cooling centrifugation to obtain different CS: TPP mass ratios. Similarly, the FITC-loaded NPs were achieved by replacing Res with a hydrophilic fluorescent probe and used as a negative control. Particle size (hydrodynamic diameter), polydispersity index (PDI), and zeta potential measurements were realized as reported in Conte et al. [29] by NanoSight NS300 Nanoparticles Tracking Analysis (NTA, Malvern Instruments, Amesbury, UK). The Res-NPs’ encapsulation efficiency (EE) was determined by detecting the amount of Res residual (described in Section 2.4.4) in the supernatant via the following equation:

(1) $$Encapsulation\;Efficiency\;\left(EE\%\right)=\frac{Total\;amount\;of\;loaded\;Res-free\;Res\;in\;supernatan}{Total\;amount\;of\;loaded\;Res}\times 100$$

### 2.3. Fabrication and Characterization of Res-Loaded Hydrogel (Res@gel)

#### 2.3.1. HA Hydrogel Preparation

Briefly, 200 mg of HA in 5 mL of double distilled water under stirring for 24 h was used to formulate the hydrogel, and then lyophilized Res-NPs (1, 5, and 10% *w*/*w* to HA) were added to HA hydrogel and stirred for an hour at 4 °C.

#### 2.3.2. Swelling Test

Dried hydrogels were submerged in a physiological saline solution (NaCl 0.9% *w*/*w*) for 24 h before being filtered out. The equation was used to determine the hydrogels’ swelling ratio (SR) (2). Three measurements were made, and the averages were calculated.

(2) $$SR=\frac{Ww}{Wd}\;$$

where W_w_ and W_d_ stand for the hydrogels’ respective wet and dry weights.

#### 2.3.3. Rheological Characterization

Rheology data were collected with a HAAKE Rheo Stress 6000 (Thermo Scientific, Milan, Italy) equipped with a parallel plate geometry, 20 mm plate diameter and 1.0 mm gap at 37 °C. Frequency sweep tests were performed at 2% strain over a frequency range of 0.1–200 rad s^−1^.

#### 2.3.4. Short-Term Stability Studies

The physicochemical stability of Res@gels was determined upon 14-day storage at different temperatures (4.0 ± 0.5 °C and 25.0 ± 0.5 °C) as reported by [27].

#### 2.3.5. In Vitro Res Release

The dialysis bag method in phosphate buffer saline was used to assess the cumulative Res release from the hydrogel formulations (PBS, pH 7.4) [30]. For this purpose, pre-swollen cellulose membrane dialysis bags (3.5–5.0 kDa cut-off, Spectrum) with the Res@gel formulations (1 mL) were immersed into 5 mL of PBS buffer (pH 7.4) in a water bath at 37 °C and shaken at 100 rpm for 5 days. Afterward, the amount of released Res in the PBS media was evaluated with liquid chromatography–tandem mass spectrometry (LC-MS/MS), as indicated by Amaghnouje et al. [31].

### 2.4. In Vitro Cell Studies

#### 2.4.1. Cell Culture and Treatment

The human keratinocyte cell line (HaCaT) was obtained from American Type Culture Collection (ATCC, Manassas, VA, USA) and cultured at 37 °C in a humidified atmosphere with 5% CO_2_ in Dulbecco’s modified Eagle’s Medium (DMEM) supplemented with 10% fetal bovine serum (FBS), 1% L-glutamine, 50 U/mL penicillin, 50 mg/mL streptomycin (Euroclone, Milan, Italy) at 37 °C in a humidified atmosphere with 5% CO_2_. Cells were analyzed for mycoplasma contamination and used at 80% confluent monolayer for all experiments. The Res@gel influence on cell proliferation was evaluated at different time points (24, 48, 72, and 96 h) by CCk8 assay as a manufacturing protocol (Euroclone, Milan, Italy). The protective effects of Res were studied in an atopic-like dermatitis model by pre-treating cells with Res@gel for 24 h and 96 h and subsequent treatment with TNF-α (10 ng/mL)/IFN-γ (10 ng/mL) for 24 h in the absence of @gels. An exposure of 4 hours was applied to evaluate the effects on mRNA expression. Cells cultured in a culture medium containing phosphate-buffered saline (PBS) in a similar amount as other treatments were used as control (CTL).

#### 2.4.2. Intracellular Oxidative Stress

Intracellular reactive oxygen species (ROS) were evaluated as reported by Di Cristo et al. [32] Pre-treated cells were labeled for 1 h in the dark at 37 °C with DCFH-DA (25 µM). Fluorescence values were collected every 5 min for 1 h using a microplate reader Cytation 3 (ASHI, Milan, Italy) with an excitation wavelength of 485 nm and an emission wavelength of 535 nm.

#### 2.4.3. Enzyme-Linked Immunosorbent Assay (ELISA)

In supernatants from HaCaT-treated cells, protein levels of secreted interleukin-4 (IL-4), interleukin-5 (IL-5), interleukin-6 (IL-6), interleukin-13 (IL-13), interleukin-25 (IL-25), interleukin-33 (IL-33), and thymic stromal lymphopoietin (TSLP) were measured [27]. The color intensity was measured using a Cytation 3 Microplate Reader at 450 nm.

#### 2.4.4. Real-Time Quantitative PCR (qRT-PCR)

TriFast (EuroClone, Milan, Italy) was used to completely extract the RNA from cell cultures following the manufacturer’s instructions, and qRT-PCR amplification was used to determine the levels of mRNA present [33]. Total RNA (0.5 µg) was processed and amplified by qRT-PCR for retro-transcription per the EuroClone standard methodology. Table 1 provides a list of specific primers for IL-4, IL-5, IL-6, IL-13, IL-25, IL-33, TSLP, and -Actin (ACTB). qRT-PCR was run on a 7900 HT fast real-time PCR System (Applied Biosystem, Milan, Italy). In addition, the reactions were performed following the manufacturer’s instructions by utilizing the SYBR Green PCR Master mix (Euroclone, Italy). The results were normalized to the housekeeping gene (ACTB), and the 2^−ΔΔC^_t_ method was performed to quantify. The results were shown as mean ±SD after each reaction was carried out in triplicate.

### 2.5. Statistical Analysis

GraphPad Prism 6 software (GraphPad Software Inc., San Diego, CA, USA) was used to compare the different experimental groups and controls statistically. All experiments were repeated at least three times, and all quantitative data are expressed as mean ± standard deviation (SD).

## 3. Results and Discussion

### 3.1. Preparation and Physicochemical Characterization of Res-Loaded Nanoparticles (Res-NPs)

Atopic dermatitis is a complex, multi-factorial pathology characterized by an impairment of the stratum corneum barrier because of decreased water content. Consequently, the skin becomes extremely dry, leading to skin lesions in response to several external stimuli (e.g., dry air, sweat, and skin microorganisms) [34]. Topical treatment is the most promising approach for managing AD because of the reduced risk of systemic side effects and the high concentration of drugs that can be achieved at the disease site [35]. Innovative nanoformulations, such as micro- and nanoparticulate systems and nanohydrogels, should exhibit the capacity to reduce adverse effects and increase local drug delivery. To reach this goal, in this work, Res-loaded chitosan nanoparticles (Res-NPs) were successfully produced, as reported by Valentino et al. [30] and then incorporated into HA hydrogels. The optimal process parameters were determined by varying the ratio of CS/TPP and the chitosan concentration, to achieve the NPs with high-Res loading and narrow polydispersity index (PDI). The particle size ranged between 563.15 ± 12.24 nm (CS: TPP 1:1) and 121.22 ± 2.43 nm (CS: TPP 10:1), demonstrating that the CS:TPP ratio strongly influences nanoparticle size (Table 2). Moreover, all formulations present a positive surface charge (from 13.91 ± 0.02 to 19.42 ± 0.11) with EE% ranging between 16.31 ± 2.23% of 1:1 and 76.18 ± 3.16% of 10:1. This is most likely due to the number of crosslinking units and the kinetics of CS crosslinking associated with different TPP concentrations, which allows increasing the rate of drug encapsulation [30].

**Figure 1:**
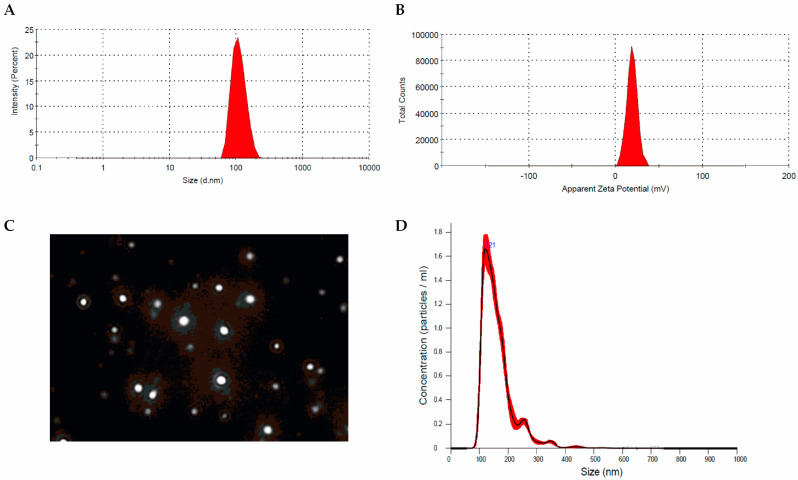
Res-NPs. (**A**) Size distribution, (**B**) zeta potential profile, (**C**) screenshot of representative NTA video, and (**D**) NTA measurements for Res-NPs in suspension. Frequency distributions are averages of 3 measurements.

Since nanoparticles obtained with a CS/TPP ratio 10:1 exhibited better EE with adequate particle size and distribution (Figure 1), this sample was chosen for further studies (Res-NPs).

Similar results were reported by Pandey et al. [36], who fabricated betamethasone-valerate-loaded CS-NPs decorated with hyaluronic acid as a promising delivery system for AD management. The prepared NPs showed a narrow size of around 300 ± 28 nm, with a positive surface charge (58 ± 8 mV) and an entrapment efficiency of up to 80%. Moreover, the amount of drug retained in the epidermis and dermis was higher in the case of decorated NPs with respect to the naked ones. In another study, Wang et al. prepared CS-NPs to improve the delivery of nicotinamide and tacrolimus with respect to commercial ointment (Protopic) [37]. The study demonstrated that the NPs increased effect through and into the skin, supported by enhanced amounts of tacrolimus in the skin. Siddique et al. formulated an aqueous cream with CS NPs to deliver hydrocortisone (a glucocorticoid for topical application) and hydroxytyrosol (a polyphenol with antioxidant properties) [38]. The cream developed by the authors showed increased safety and tolerability without systemic effects or toxicity.

### 3.2. In Vitro Hydrogel Formulation (Res@gel) and Res Release

**Figure 2:**
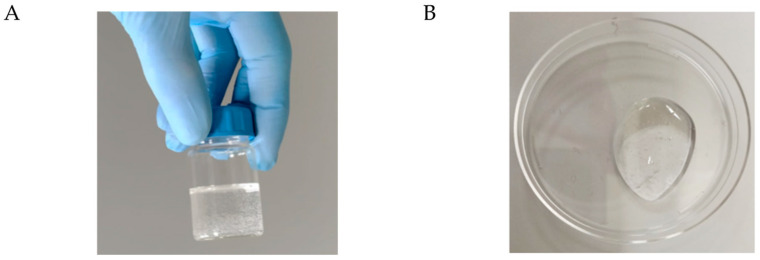

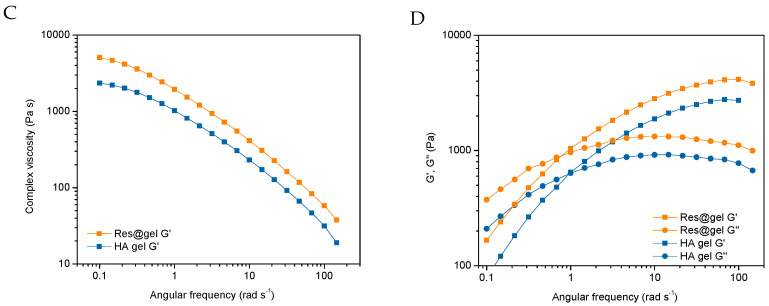
Appearance and rheological properties of the HA-based hydrogels. (**A**,**B**) Representative images of Res@gel_10_. Dependence of (**C**) viscosity and (**D**) viscoelastic on the angular frequency of HA and Res@gel_10_.

Several studies reported that HA-based transdermal delivery systems exhibit excellent biocompatibility, enhanced permeability, and efficient localized release of topical anti-inflammatory drugs, inhibiting eczema-associated skin inflammation [39]. Moreover, the bioactive effect of hyaluronic acid in filling the intercellular spaces of the living epidermal layers is well known, highlighting its role in the treatment of AD pathology [40]. These properties, alongside the enhanced controlled release abilities, improved targeting capacity to the skin strata, and easy manipulation of swelling level [41], make HA gels efficient devices for topical drug delivery. The protective effect of gel structures against nanoparticle aggregation and clustering supported these advantages [42]. Hence, the delivery of resveratrol on the dermal target site for AD treatment was further ameliorated by incorporating Res-NPs into a hyaluronic acid gel (Res@gel). The hydrogels were prepared using 4% wt of polymer with different nanoparticle concentrations. Res@gel showed smooth consistency and was homogeneous and transparent (Figure 2A,B). The viscoelastic properties of the HA-based gels were assessed by oscillatory rheology. First, the range of linear viscoelasticity of the HA-based formulations was assessed, and a value well above 10% strain was noted for HA and Res@gel containing 10% Res-NPs (Figure S1). Then, frequency sweep tests carried out at 2% strain provided information on the evolution of viscosity, viscous (G″), and storage (G′) moduli as a function of frequency (Figure 2C,D). For HA, the viscosity vs. angular frequency curves shows the material’s pseudoplastic nature and shear-thinning behavior (Figure 2C). Moreover, a dependence of G′ was observed, and the transition from a liquid-like (G″ > G) to a solid-like (G′ > G″) behavior occurred at a very low angular frequency (about 1 rad s^−1^) corresponding to a value of about 600 Pa (Figure 2D). That is, a stiff behavior was noted due to the high molecular weight of HA. The dependence of G″ on the frequency was lower than G′, achieving a maximum value of 900 Pa at 10 rad s^−1^, further confirming the large amount of energy required to deform the material. The addition of 10% Res-NPs did not significantly affect the HA structure, since the rheological curves exhibited a comparable trend. However, Res@gel displayed higher viscosity and moduli values throughout the investigated frequency range, suggesting that the presence of the nanoparticles was able to tighten the interactions between HA chains, resulting in a stiffer material.

As previously mentioned, the ameliorative action of the gel structure on the bioactivity of compounds encapsulated in nanovehicles has already been assessed. For example, Duarah et al. formulated vitamin C ethyl cellulose nanoparticles dispersed in hydroxypropyl methylcellulose gels to prevent oxidative damage to the skin. Such preparations exhibited a sustained release over 8 h with improved ascorbic acid concentration at the application site [43]. Similarly, Elmowafy et al. produced indomethacin nanoparticles composed of poly(ɛ-caprolactone) and hydroxypropyl β-cyclodextrin, mixing them into a methylcellulose and Carbopol 940 gel. They obtained higher encapsulation efficiency with an improved release on the site of action [44]. Hatem et al. [45], confirmed the efficacy of these systems in topical delivery by using functionalized chitosan nanoparticles in gel form to improve the local delivery of alpha-arbutin as a skin-bleaching agent for melisma treatment. Furthermore, chitosan nanoparticles showed great stability and showed continued release of α-arbutin over 24 h. The ex vivo deposition study indicated the excellence of chitosan nanoparticles in concentrating the drug in the inner skin layers to avoid transdermal delivery. Jana et al. prepared aceclofenac-loaded chitosan/egg albumin nanoparticles dispersed in a Carbopol 940 gel for transdermal drug delivery. The in vitro assay demonstrated sustained drug release over 8 h with enhanced drug efficiency. The in vivo anti-inflammatory effect in carrageenan-induced rats revealed that the formulated gel inhibited swelling of rat paw edema more effectively than commercial aceclofenac gel [46].

**Figure 3:**
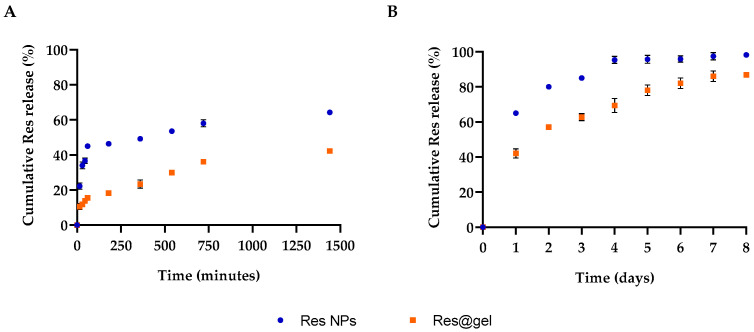
Cumulative Res release from Res NPs and Res@gel_10_ in phosphate buffer saline (PBS) after (**A**) 24 h and (**B**) 8 days. Six different experiments were performed, and the results were expressed as the mean of the obtained values (mean ± SD).

Then, the improved efficiency of the gel system in delivering bioactive nanoparticles was assessed in vitro, evaluating resveratrol release from Res-NPs and Res@gel with a dialysis membrane against phosphate buffer saline (PBS). As reported in Figure 3, the Res release kinetics are characterized by a rapid release rate of 45% during the first hour, followed by a sustainable and gradual release for 2 days (80%). Meanwhile, the Res releasing rate from Res@gel significantly decreased (*p* < 0.05), and only 15% of Res was released after 1 h; subsequently, a steady Res release for up to 1 week was observed and reached 80% after 5 days. The slow Res release from the hydrogel may be associated with the viscosity of the hydrogel structure. The aggregated polymeric chains form a packed hydrated matrix that hampers nanoparticle migration and explains the role of HA as a structural component of the ECM [47,48].

Moreover, the protective effect of the Res@gel formulation on the physical stability of the chitosan nanoparticles was investigated through 4 weeks of assessment of the size and PDI of Res NPS released from Res@gel. As reported in Table 3, protected Res NPs, differently from their free form, were stable when stored at 4 and 25 °C without significant particle size and PDI increase (Figure S2). In addition, the pH of the formulation (pH 6.8) remained unaltered throughout the storage time at both temperatures considered.

### 3.3. Antioxidant Activity of Res@gel_10_ in AD-Induced Cellular Model

The skin is a major oxidative stress target. Indeed, reactive oxygen species (ROS), reactive nitrogen species (RNS), and reactive metabolites are continually generated in response to environmental and endogenous pro-oxidant agents in the keratinocytes. While free radicals produced during normal metabolism are intrinsic to normal skin function, prolonged ROS action can overwhelm the skin’s antioxidant defense mechanisms and contribute to skin disorders such as skin cancer, skin aging, and dermatitis. In addition, the resulting dermal inflammation affects the efficiency of the skin barrier, allowing microbial colonization [49]. Although few clinical studies investigated the relationship between AD treatments and oxidative stress, it was highlighted that the intake of antioxidants ameliorated patients’ conditions [50].

**Figure 4:**
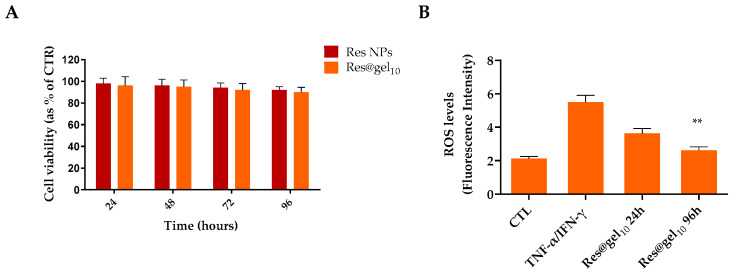
(**A**) Effect of Res@gel_10_ on cell proliferation after 24, 48, 72, and 96 h. Untreated cells were used as control (CTL). (**B**) The antioxidant capacity of Res@gel_10_ in TNF-α/IFN-γ-treated HaCat cells was evaluated by oxidized H2DCFDA (DCF). The cells were previously incubated for 24 and 96 h with Res@gel_10_ and then stimulated with TNF-α/IFN-γ for 24 h. Results are expressed as the mean of three independent experiments ± S.D (n = 3). ** *p* < 0.01 versus CTL.

The safety of HA and CS is widely recognized due to their biocompatibility and biodegradability, although their nanosized counterparts may be toxic. Hence, to assess the safety of nano-based formulations, a cytotoxicity assay of Res-NPs and Res@gel_10_ was performed in human keratinocytes over 96 h. As reported in Figure 4A, both formulations did not exert toxic effects on cell proliferation until 96 h, confirming that Res-NPs and Res@gel_10_ show an acceptable level of biocompatibility.

Among the naturally bioactive molecules, Res has been studied mainly for its radical scavenger activity both in vivo and in vitro, demonstrating its capability to regulate mitochondrial dysfunction and eliminate reactive oxygen species [51,52]. In fact, owing to its anti-inflammatory and antioxidant activity, Res has been investigated as a natural alternative for the treatment of several skin disorders, including AD [52].

To investigate the therapeutic potential of Res@gel_10_ in the context of AD, human keratinocytes HaCaT were stimulated with TNF-α/IFN-γ inducing the production of cellular and mitochondrial ROS and the secretion of proinflammatory cytokines, simulating the in vivo condition typical of AD. As shown in Figure 4B, HaCaT treatment induces a four-fold increase in ROS production compared to untreated cells used as control (CTL). Interestingly, when the cells were pre-treated with Res@gel_10_, ROS-generated levels decreased by about 1.5-fold concerning TNF-α/IFN-γ cells after 24 h. In addition, a longer pre-treatment (96 h) significantly increased the protective effect of released Res (*p* < 0.001), resulting in a slight fluorescence increase in comparison to control cells.

### 3.4. Inflammatory Potential of Res@gel_10_

**Figure 5:**
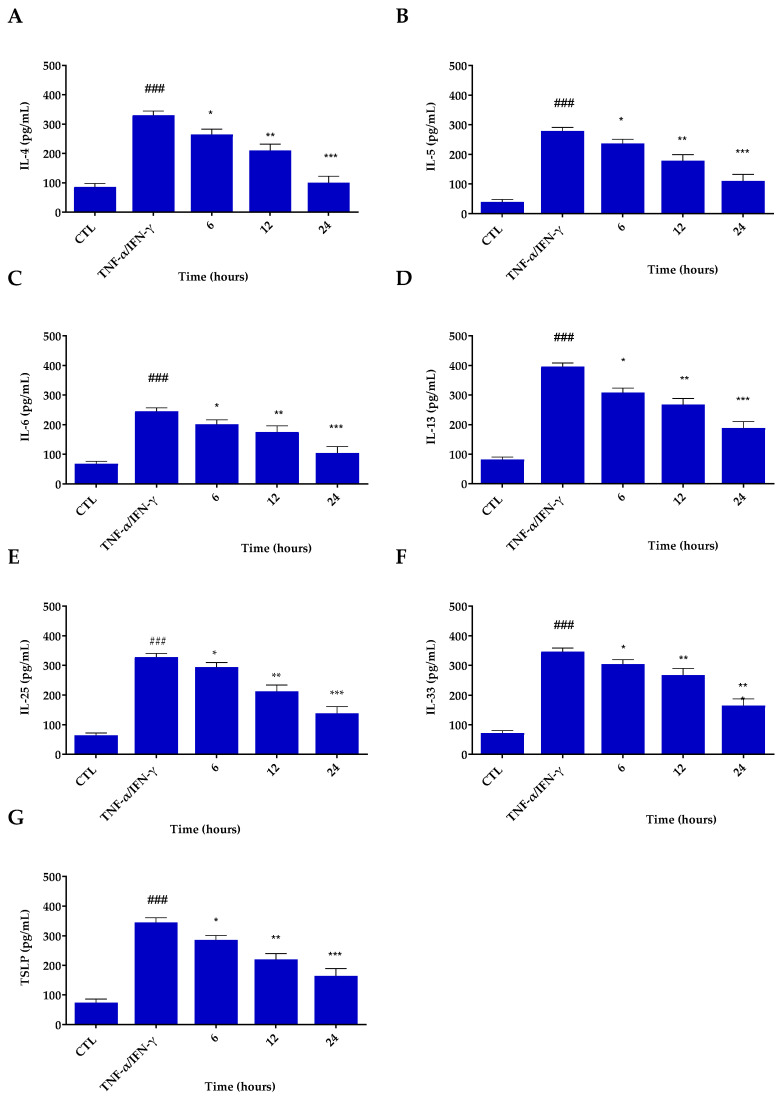
Inhibitory effects of Res@gel_10_ on inflammatory cytokine secretion in TNF-α/INF-γ-induced HaCaT cells. Secretion of IL-4 (**A**), IL-5 (**B**), IL-6 (**C**), IL-13 (**D**), IL-25 (**E**), IL-33 (**F**), and TSLP (**G**) was measured by ELISA assay. Cells were pre-treated with Res@gel_10_ for 24 h, then stimulated with TNF-α/IFN-γ for 24 h. Results are expressed as the mean of three independent experiments ± S.D (n = 3). ### *p* < 0.001 TNF-α/IFN-γ-treated cells vs. CTL, * *p* < 0.05, ** *p* < 0.01, and *** *p* < 0.001 Res@gel_10_ vs. TNF-α/IFN-γ-treated cells.

AD is an inflammatory skin disease accompanied by a severe itching sensation and epidermal barrier dysfunction. In the current study, the ability of Res@gel_10_ to modulate the inflammatory cytokine secretion in TNF-α/IFN-γ-stimulated HaCaT cells was assessed by enzyme-linked immunosorbent assays (ELISA) and quantitative real-time PCR (qRT-PCR) analysis. As expected, when keratinocytes are exposed to TNF-α/IFN-γ, chemokine expression significantly increases (*p* < 0.001) with respect to untreated cells used as control (Figure 5). Res@gel_10_ pre-treatment substantially exerts an inhibitory effect on the secretion of all examined proinflammatory cytokines compared to those in cells treated with TNF-α/IFN-γ alone. As shown in Figure 5, Res activity is already evident after 6 h and becomes significantly more noticeable after 24 h (*p* < 0.001).

Several studies report evidence that pre- and post-treatment with resveratrol has promising potential for treating inflammatory diseases such as AD [53,54,55]. Res was shown to be bioactive in a murine model of 2,4-dinitrophenylbenzene (DNFB)-induced AD-like lesions by Sozmen et al. Res decreased inflammation and histological changes, affecting apoptosis and regulating the secretion of cytokines in the epithelium [56].

In another work, Shein and Xu proved that Res ameliorated AD-like skin lesions in BALB/c mice. Dermal treatment with Res slowed the progression of AD-like skin lesions and ameliorated DNCB-induced dermal destruction in mice, reducing the levels of proinflammatory cytokines. Moreover, Res treatment led to the upregulation of several cytokines, such as filaggrin (FLG), envoplakin (EVPL), and transglutaminase (TG) [57].

Keratinocytes form a multilayered structure with the priority function of maintaining the skin’s functional integrity. AD patients exhibit an increased production of cytokines and chemokines by keratinocytes, demonstrating their involvement in the pathophysiology of AD. In reply to dermal barrier injury, keratinocytes secreted proteins such as thymic stromal lymphopoietin (TSLP), interleukin-25 (IL-25), and IL-33, which activated OX40L/OX40 (TNF family members) signaling, leading to type 2 immune deviation. OX40-stimulated Th2 cells expressed IL-4, IL-5, IL-6, and IL-13 [58,59]. IL-4 drives TH2 cell differentiation and IgE class switching in B lymphocytes, while IL-13 is involved in B-cell maturation, differentiation, and eosinophil chemotaxis. Further, Th2-derived IL-13 and IL-4 reduce the expression of skin-barrier-related proteins such as filaggrin and loricrin and exacerbate epidermal barrier dysfunction, resulting in Staphylococcus aureus colonization and increased penetration of external allergens [60].

**Figure 6:**
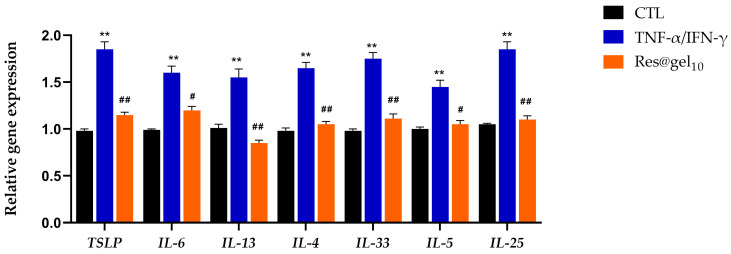
In AD-like induced HaCaT cells, Res@gel10 affects the mRNA levels of inflammatory cytokines. Cells were pre-treated with Res@gel_10_ for 24 h and stimulated with TNF-α/IFN-γ for 4 h. Results are expressed as the mean of three independent experiments ± S.D (n = 3). # *p* < 0.05, and ## *p* < 0.01 TNF-α/IFN-γ-treated cells vs. CTL, ** *p* < 0.01 Res@gel_10_ vs. TNF-α/IFN-γ-treated cells.

As shown in Figure 6, RT-qPCR results corroborated that cytokine and chemokine mRNA levels were significantly increased in TNF-α/IFN-γ-treated cells compared to the untreated group (*p* < 0.001). Res@gel_10_ pre-treatment consistently reduced the mRNA levels of all tested cytokines (relative to the housekeeping gene), with about 50% reduction concerning TNF-α/IFN-γ-induced HaCaT cells.

These results demonstrated the ability of Res@gel_10_ to exert an efficient anti-inflammatory effect through the regulation of both secretion and expression of proinflammatory cytokines in TNF-α/INF-γ-induced human keratinocytes.

## 4. Conclusions

Atopic dermatitis is a highly prevalent skin disease that occurs in early childhood and may persist into adulthood. The condition has a chronic course and can significantly impact patients’ quality of life. It is a multifactorial pathology whose causes are not clearly understood. However, it is assumed that inflammatory and oxidant stress are responsible for AD symptomatology. Resveratrol has excellent anti-inflammatory and antioxidant activities. However, its poor bioavailability requires a drug delivery system to perform its properties better. This work supported new insights into the therapeutic effects of applying Res-loaded chitosan nanoparticles embedded in hyaluronic hydrogels (Res@gel) as an adjuvant in the treatment of AD symptomatology; 120 nm-sized crosslinked CS nanoparticles were able to encapsulate up to 80% Res, and the HA matrix protected them from hydrolytic degradation, also retarding Res release. Synthesized Res@gel had no effect on keratinocyte proliferation, demonstrating good biocompatibility. Res released from hydrogel significantly reversed ROS production from TNF-α/IFN-γ-treated keratinocytes used as an AD in vitro model. In addition, pre-treatment with Res@gel decreased secretion and expression of proinflammatory cytokines such as IL-4, IL-6, and IL-33, known to be upregulated in AD disease. Hence, the formulated hydrogel is proposed for efficient and sustained Res topical delivery for AD treatment.

## Figures and Tables

**Table 1:** Primers used for qRT-PCR.

Gene	Accession Number	Forward	Reverse
*IL-4*	NM_000589.4	ACTGCACAGCAGTTCCACAG	CTCTGGTTGGCTTCCTTCAC
*IL-5*	NM_000879.3	TGAGGATGCTTCTGCATTTG	GCAGTGCCAAGGTCTCTTTC
*IL-6*	NM_000600.5	CGCCTTCGGTCCAGTTGCC	GCCAGTGCCTCTTTGCTGCTTT
*IL-13*	NM_002188.3	CATCGAGAAGACCCAGAGGA	TTTACAAACTGGGCCACCTC
*IL-25*	NM_022789.4	GGACTCCTAACCTGCTCCAG	CTCTGCACTGACCTGGTACA
*IL-33*	NM_033439.4	CAAAGAAGTTTGCCCCATGT	AAGGCCTTTTGGTGGTTTCT
*TSLP*	NM_033035.5	ATGAGAGGCAAAACCTGGTG	AATTCCACCCCAGTTTCACA
*ACTB*	NM_001101.5	ACTCTTCCAGCCTTCCTTCC	CGTACAGGTCTTTGCGGATG

**Table 2:** Effect of chitosan concentration and chitosan/TPP ratio on the size (hydrodynamic diameter), polydispersity index (PDI), zeta potential (ZP), and encapsulation efficiency (EE) of Res-loaded nanoparticles (Res-NPs). The Res concentration was held constant at 10 mg.

Chitosan (mg/mL)	CS:TPP Mass Ratio	Size (nm ± SD)	PDI (nm ± SD)	Z Potential (mV ± SD)	Encapsulation Efficiency (%±SD)
0.1	1:1	563.15 ± 12.24	0.37 ± 0.04	13.9 ± 0.02	16.31 ± 2.23
0.1	5:1	352.39 ± 8.19_a_	0.28 ± 0.03	12.7 ± 0.01	39.4 ± 1.31_a_
0.1	10:1	247.26 ± 4.21_b_	0.19 ± 0.04	13.5 ± 0.02	41.5 ± 2.23_a_
0.5	1:1	514.24 ± 10.13	0.31 ± 0.03	15.3 ± 0.07	23.7 ± 1.45_b_
0.5	5:1	338.26 ± 7.49_a_	0.33 ± 0.02	16.9 ± 0.02	46.7 ± 1.12_a_
0.5	10:1	177.15 ± 3.12_c_	0.22 ± 0.04	16.1 ± 0.03	55.8 ± 1.64
1	1:1	469.31 ± 9.78	0.25 ± 0.03	17.5 ± 0.07	30.3 ± 1.91_b_
1	5:1	315.17 ± 7.21_b_	0.21 ± 0.02	18.6 ± 0.09	59.9 ± 2.26
1	10:1	121.22 ± 2.43_c_	0.24 ± 0.04	19.4 ± 0.1	76.18 ± 3.16

Note: Values with the same subscript letter (a–c) in the same column were not significantly different (*p* > 0.05). Data were mean of three independent experiments ± standard deviation (SD).

**Table 3:** Nanoparticle stability studies.

	Res-NPs before Storage	Free Res-NPs	Res-NPs Released from Res@gel at 4 °C (nm ± SD)	Res-NPs Released from Res@gel at 25 °C (nm ± SD)
Average particle size	123.57 ± 9.11	269.18 ± 19.24	135.64 ± 9.04	141.77 ± 10.21
PDI	0.15 ± 0.03	0.17 ± 0.05	0.11 ± 0.03	0.19 ± 0.04

## Data Availability

The data presented in this study are available in the article.

## Supplementary Materials

The following supporting information can be downloaded at: https://www.mdpi.com/article/10.3390/jfb14020082/s1, Figure S1: Dependence of viscoelastic moduli on the angular frequency; Figure S2: Size distribution of Res NPs before and after stability studies.
