# Supplementary material for: Hyaluronic Acid Hydrogel Containing Resveratrol-Loaded Chitosan Nanoparticles as an Adjuvant in Atopic Dermatitis Treatment
Source: J Funct Biomater. 2023 Jan 31;14(2):82. doi: 10.3390/jfb14020082 (PMC9959248; doi:10.3390/jfb14020082)
Supplement: Supplementary file 1 [file jfb-14-00082-s001.zip › jfb-2136512-supplementary.pdf]

Supplementary Data

Hyaluronic acid hydrogel containing resveratrol-loaded chitosan nanoparticles as an adjuvant in atopic dermatitis treatment

Raffaele Conte[1#], Ilenia De Luca[2#], Anna Valentino[2], Pierfrancesco Cerruti[3], Parisa Pedram[4], Gustavo Cabrera-Barjas[5], Arash Moeini[4,*] and Anna Calarco[2,*]

1 AMES Group Polydiagnostic Center, via Padre Carmine Fico, 24, 80013 Casalnuovo di Naples, Italy
2 Research Institute on Terrestrial Ecosystems (IRET)—CNR, Via Pietro Castellino 111, 80131 Naples, Italy
3 Institute of Polymers, Composites and Biomaterials (IPCB) – CNR, Via Campi Flegrei, 34, 80078 Pozzuoli NA, Italy
4 Technische Universität München (TUM) School of Life Sciences Lehrstuhl für Brau- und Getränketechnologie, 85354 Freising, Germany
5 Universidad de Concepción, Unidad de Desarrollo Tecnológico, UDT, Avda. Cordillera No. 2634, Parque Industrial Coronel, Coronel, Chile
* Correspondence: arash.moeini@tum.de (A.M.); anna.calarco@cnr.it (A.C.)
[#] These authors contributed equally to this work.

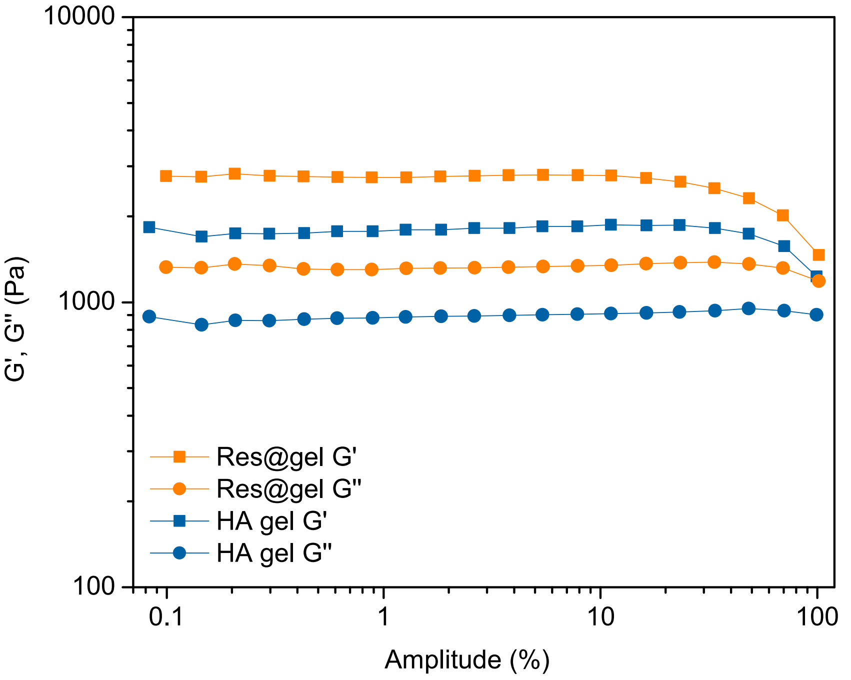

Figure S1. Dependence of viscoelastic moduli on the angular strain.

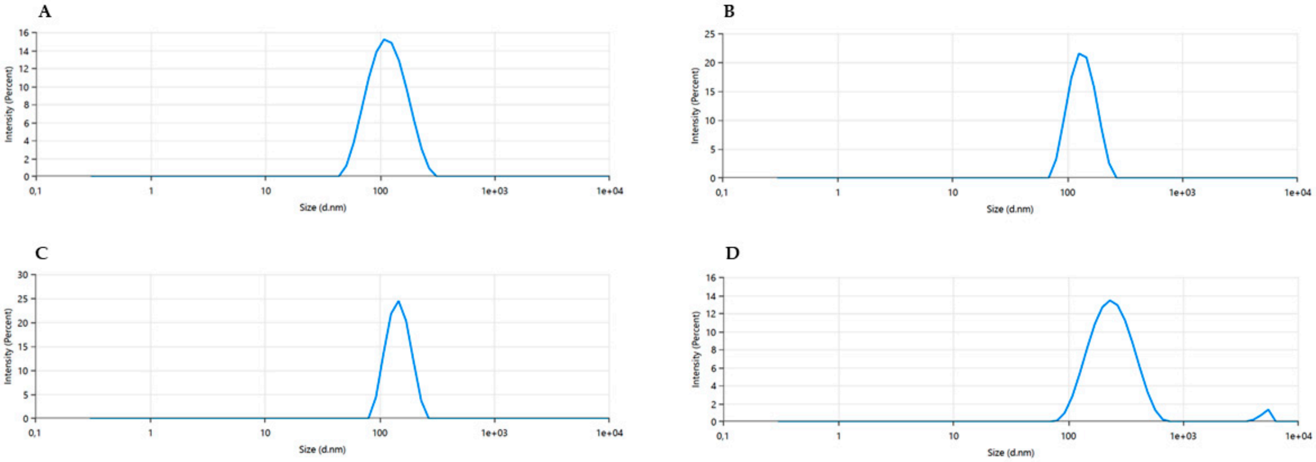

Figure S2. Particles PDI.
